# Zika virus infection in the returning traveller: what every neurologist should know

**DOI:** 10.1136/practneurol-2017-001789

**Published:** 2018-04-04

**Authors:** Sonja Emily Leonhard, Suzannah Lant, Bart C Jacobs, Annelies Wilder-Smith, Maria Lucia Brito Ferreira, Tom Solomon, Hugh John Willison

**Affiliations:** 1 Department of Neurology, Erasmus MC, University Medical Center, Rotterdam, The Netherlands; 2 Institute of Infection and Global Health, The University of Liverpool, Liverpool, UK; 3 Department of Neurology and Immunology, Erasmus MC, University Medical Center, Rotterdam, The Netherlands; 4 Unit of Epidemiology and Global Health, Department of Public Health and Clinical Medicine, Umeå University, Umeå, Sweden; 5 Department of Neurology, Hospital da Restauração, Recife, Brazil; 6 National Institute for Health Research Health Protection Research Unit in Emerging and Zoonotic Infections, University of Liverpool, Liverpool, UK; 7 Department of Neurology, Walton Centre NHS Foundation Trust, Liverpool, UK; 8 Department of Neurology and Institute of Infection, Immunity and Inflammation, University of Glasgow, Glasgow, UK

**Keywords:** zika virus, neurovirology, clinical neurology, Guillain-Barré syndrome, neuroimmunology

## Abstract

Zika virus has been associated with a wide range of neurological complications. Neurologists in areas without current active transmission of the virus may be confronted with Zika-associated neurological disease, as a large number of returning travellers with Zika virus infection have been reported and the virus continues to spread to previously unaffected regions. This review provides an overview of Zika virus-associated neurological disease and aims to support neurologists who may encounter patients returning from endemic areas.

## Introduction

The exponential increase in travelling has major consequences for the epidemiology of infectious and postinfectious disease, constituting a global public health challenge.^1^ The most recent example of this is the Zika virus (ZIKV) epidemic in Latin America that has now spread to 84 countries globally and has been associated with severe neurological sequelae, including microcephaly, Guillain-Barré syndrome (GBS) and central nervous system (CNS) disorders.^2^ Regions without active transmission, including Europe, East Asia and North America, should still be on high alert, as large numbers of returning travellers with ZIKV infection have been reported and parts of these regions are predicted to be at risk for active viral transmission during the summer months.^3–5^ This review aims to support neurologists who may see travellers with neurological complications returning from ZIKV endemic areas.

### Transmission

**Figure 1:**
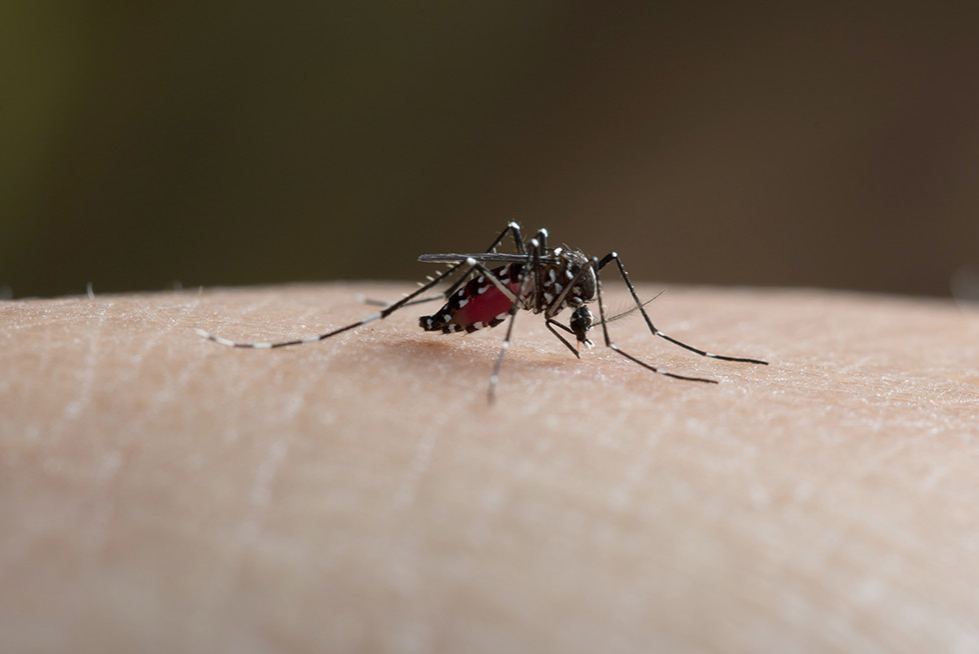
*Aedes* mosquito.

ZIKV is an enveloped positive-strand RNA member of the *Flavivirus* genus in the Flaviviridae family. Other flaviviruses include dengue, yellow fever, West Nile virus and Japanese encephalitis virus, many of which are associated with neurological disease.^6^ Like these viruses, ZIKV is principally transmitted by a mosquito bite and is thus described as an arthropodborne virus or ‘arbovirus’. Its most prolific vector is the *Aedes aegypti* mosquito, which transmits the virus between humans and is widespread in tropical regions globally (figure 1). Other *Aedes* mosquitos that populate more temperate regions, such as *Aedes albopictus*, can also facilitate viral spread but do so less effectively.^7^

Direct transmission between humans can occur vertically from mother to fetus, via blood transfusion, and by sexual contact; the latter accounts for an estimated 1% of ZIKV cases in Europe.^8 9^ Thus, while human-to-human transmission is far less common than conventional arboviral transmission, it remains important to elicit a history of potential exposure to contacts that might have led to subsequent infection.

### Geographical spread

ZIKV derives its name from the Zika forest in Uganda where it was identified in 1947. The first outbreak was reported in Micronesia in 2007, 60 years after its discovery. The link between ZIKV and neurological complications was first recognised when an epidemic in French Polynesia in 2013 was followed by a 20-fold increase in incidence of GBS cases.^10^ It is unclear if a change in viral strain altered its pathogenicity, or whether the high incidence of infection resulted in a noticeably large number of otherwise rare neurological manifestations. Recent data suggest a mutation in the virus’s non-structural 1 protein, occurring around 2013, might promote the acquisition of the virus by its mosquito vector, thus enhancing its transmission in recent epidemics.^11^

Cases were next reported in Brazil in early 2015, where the virus went on to affect an estimated 0.5–1.5 million people. During these months, there were alarming increases of reported microcephaly and GBS cases, prompting the WHO to declare ZIKV a Public Health Emergency of International Concern on 1 February 2016. The virus has since spread to a further 84 countries in the Americas, Africa and Asia, including 225 cases through presumed local mosquitoborne transmission in Southern states of the USA.^2^

**Figure 2:**
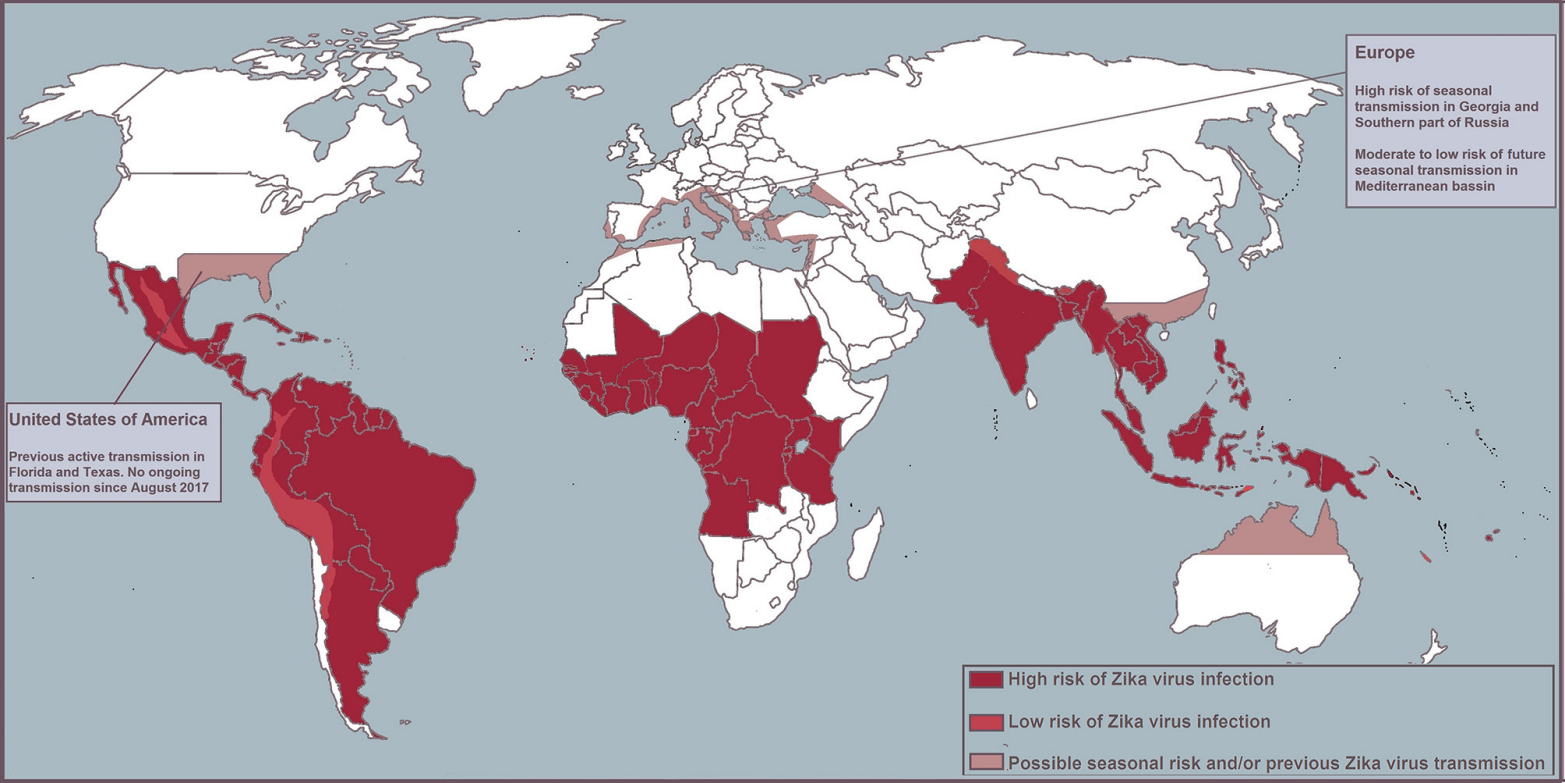
Map of high- and low-risk areas for infection.

Despite the decreasing trend of ZIKV infection in the Americas in 2017, the global health community advocates vigilance as it is unclear where and when the next outbreak will take place. Models predict that South and Southeast Asia and Oceania are at high risk for future outbreaks, with seasonal transmission a possible threat in southern parts of North America, China and Europe^3 4^ (figure 2).

While there has been no local spread reported in Europe and transmission has ceased in North America, there have been approximately 2130 travel-imported cases in Europe and around 5500 travel-imported cases in the USA since the start of the Brazilian epidemic.^12 13^ In clinical practice, suspicion of ZIKV or related arboviral infections, including dengue and chikungunya virus, should be high in anyone returning from—or in close contact with people returning from—endemic areas. We recommend checking the websites of Public Health England, WHO, National Travel Health Network and Centre, Centers for Disease Control and Prevention, or European Centre for Disease Prevention and Control for the latest updates regarding ZIKV spread and travel advice (box 1).

Box 1 **Useful links**

- Public Health England https://www.gov.uk/government/collections/zika-virus-zikv-clinical-and-travel-guidance.
- WHO http://www.who.int/emergencies/zika-virus/en/.
- Centers for Disease Control and Prevention https://www.cdc.gov/zika/.
- European Centre for Disease Prevention and Control https://ecdc.europa.eu/en/zika-virus-infection.
- The National Travel Health Network and Centre https://travelhealthpro.org.uk/countries.

### Systemic symptoms

ZIKV has an estimated incubation period of 3–10 days and can remain asymptomatic in approximately 80% of cases.^14^ Therefore, the absence of a febrile illness history does not exclude the diagnosis. Symptomatic infection is characterised by fever, rash, non-purulent conjunctivitis and arthralgia lasting up to a week, but may often also present as only a rash without fever or other accompanying signs and symptoms.^14^ These symptoms resemble those of other vectorborne viruses such as dengue and chikungunya virus, although a mild disease course with conjunctivitis is more specific for ZIKV (table 1). As such, it is the neurological features, rather than the systemic ones, that are the main cause of disability and death.

**Table 1:** Clinical features of arboviruses

	Zika virus	Dengue virus	Chikungunya virus
Fever	++	+++	+++
Rash (maculopapular)	+++	+	++
Conjunctivitis	++	−	−
Arthralgia	++	+	+++
Myalgia	+	++	+
Headache	+	++	++
Shock	−	+	+/−
Haemorrhage	−	+	−

Reproduced from the Centers for Disease Control and Prevention.^39^

## Neurological complications of ZIKV in the adult

ZIKV is known to be neurotropic: in neural progenitor cells infection it halts proliferation and may induce cell death, which is the likely disease mechanism of ZIKV-related microcephaly cases.^15 16^ Beyond congenital Zika, direct viral invasion as well as a parainfective or postinfective autoimmune response may contribute to pathogenesis. Box 2 lists the wide range of neurological problems associated with acute ZIKV infection.^17^

Box 2 **Neurological conditions associated with Zika**

- Congenital Zika syndrome.
- Peripheral nervous system*:
  - Guillain-Barré syndrome.
  - Chronic inflammatory demyelinating polyneuropathy.
  - Acute transient polyneuritis.
- Central nervous system*:
  - (Meningo)encephalitis.
  - Myelitis.
  - Acute disseminated encephalomyelitis (ADEM).
  - Encephalopathy.

*There have also been some cases of combined central and peripheral disease.^17^

As such, any traveller returning from an area with ZIKV transmission, or with a sexual partner who has returned from such an area, who develops an acute neurological illness should be screened for ZIKV infection. Other cocirculating arboviruses should also be considered, such as chikungunya and dengue virus, which have been linked to neurological complications.^6 17^

### Guillain-Barré syndrome

Since the start of the global ZIKV epidemic, 23 countries have reported an increased incidence in GBS in parallel with the rising incidence of ZIKV infection. An association between GBS and ZIKV was first established in a case–control study in French Polynesia and many case reports and series have since substantially linked ZIKV and GBS.^2 10 18^ Although there is no proven causality, the most likely explanation is that ZIKV infection can trigger GBS. ZIKV is not being currently actively being transmitted in Europe, but neurologists may well be confronted with GBS cases following patient travel to areas with ongoing ZIKV transmission (box 3).

Box 3 **Guillain-Barré syndrome in a returning traveller^40^**

 A 60-year-old woman who had returned to the Netherlands from Suriname 10 days before presented with diarrhoea, low-grade fever, nausea and vomiting, and an unsteady gait. Over 4 days, she developed bilateral facial palsy, symmetrical paresis of all limbs and distal hypoesthesia with low to absent tendon reflexes. Cerebrospinal fluid (CSF) had a normal cell count and increased protein concentration; electrophysiology showed signs of a demyelinating polyneuropathy. Her urine and blood were positive for Zika virus (ZIKV) RNA, and serology for other recent infections associated with Guillain-Barré syndrome (GBS) was negative. She received a 5-day course of intravenous immunoglobulin (0.4 g/L daily). At 1-year follow-up she was limited in her daily activities by fatigue and minor weakness in the legs but could walk without aid and had returned to part-time work.

GBS is an acute polyradiculoneuropathy and its classic form is characterised by a rapidly progressive symmetrical weakness of muscles in legs and arms with sensory symptoms and reduced tendon reflexes.^19^ However, the clinical presentation, disease progression and outcome may vary extensively between patients, complicating the diagnosis and treatment. Several variants of GBS have been identified, including the pure motor and paraparetic variants and the Miller Fisher syndrome, characterised by ophthalmoplegia, ataxia and areflexia. Two-thirds of GBS cases report symptoms of an infective disease in the month before disease onset, and several pathogens have been associated with GBS, including *Campylobacter jejuni*, cytomegalovirus, hepatitis E virus, *Mycoplasma pneumoniae* and Epstein-Barr virus. ZIKV is the latest pathogen to be added to this list. The current paradigm is that in GBS the preceding infection triggers an immune response that causes nerve injury. This has been best described in cases with preceding *C. jejuni* infection in which there is carbohydrate mimicry between lipo-oligosaccharides on *C. jejuni* and gangliosides on human peripheral nerves and cross-reactive antibodies to these structures.^20^ Some antibodies are associated with clinical variants or electrophysiological subtypes of GBS, reflecting in part the distribution of gangliosides in peripheral nerves. For instance, the ganglioside GM1 is predominantly present on axons and the presence of antibodies to GM1 is associated with the pure motor and axonal forms of GBS.^21^ It is not yet clear how ZIKV (and viral infections in general) can lead to GBS and no particular autoantibody biomarkers that aid in diagnosis have been identified. ZIKV might cause nerve damage by direct infection, as some patients have reported onset of GBS shortly after or even concurrent with infective symptoms, and many patients with GBS have ZIKV RNA in cerebrospinal fluid (CSF) or serum, indicating ongoing infection.^22^ However, many other reported cases have not had these features, and a recent mouse model study showed resistance of the peripheral nervous system to infection by ZIKV, indicating that an immune-mediated disease mechanism is more likely.^23^

ZIKV does not seem to be associated with a specific GBS phenotype, although most cases have a sensorimotor variant with facial nerve palsy, ventilatory insufficiency and a demyelinating pattern on clinical neurophysiology. There have also been more reported cases with paraparesis and intact reflexes than expected based on previous studies.^10 18^ It is not yet clear how to interpret these data. They could be a first indication of a specific clinical phenotype of GBS, but could also reflect the cases being labelled as GBS when in fact they result from CNS pathology. At present, there are no indications that clinicians should deviate from standard diagnostic criteria for GBS in a suspected ZIKV-related GBS case.

### Other peripheral nervous system manifestations

There have been sporadic case reports linking other peripheral nerve and neuromuscular diseases to ZIKV infection, including two cases with acute onset of chronic inflammatory demyelinating polyneuropathy (CIDP), three cases of transient polyneuritis and two cases of myasthenia gravis.^24–26^ The patients with transient polyneuritis had mild distal sensory and motor symptoms that resolved within 10 days and a positive PCR test for ZIKV in serum and CSF, suggesting a self-limiting direct peripheral nerve infection by ZIKV.

It is not yet clear if these reported cases are mere coincidence or if ZIKV is indeed a causal factor but, in patients with flaccid paralysis following ZIKV infection, clinicians should consider these differential diagnoses. Furthermore, patients diagnosed with GBS after ZIKV need careful follow-up as it may transpire that they have acute-onset CIDP.^26^

### Meningitis and encephalitis

Inflammation of the meninges (meningitis) or brain parenchyma (encephalitis) may result from viral infection of the brain. There has been no robust study associating ZIKV with CNS disease. However, meningoencephalitis in the context of recent ZIKV infection following foreign travel was first reported in early 2016 when, 10 days after returning from a cruise around New Caledonia, a patient who developed focal neurological symptoms and a concurrent rash was found to have ZIKV reverse transcription (RT)-PCR positive CSF.^27^

ZIKV-associated encephalitis is associated with a range of clinical outcomes, from full recovery to death.^28 29^ It is not yet known whether age or a compromised immune system predispose individuals to neurological complications of ZIKV infection. There may be arboviral systemic symptoms at the time of onset of neurological symptoms, encompassing confusion; impairment of memory, attention and processing speed; seizures; and focal motor deficits. Pathological changes on electroencephalography and MRI may be either diffuse or more focal. Currently there are limited data, but by analogy with other flaviviruses, ZIKV might be expected to cause high signal intensities in the thalamus and other basal ganglia.^30^

### Myelitis

Myelitis is a spinal cord inflammation that often occurs following infection and may appear in isolation, as part of a spectrum of inflammatory nervous system pathology or in multisystem disease. Patients typically present with rapid-onset motor and sensory changes that are usually bilateral and associated with a defined sensory level, as with transverse lesions. Autonomic dysfunction is common and CSF or MRI may give evidence of an inflammatory myelopathy.^31^

In contrast, some neurotropic viruses, particularly enteroviruses such as polio and flaviviruses such as Japanese encephalitis virus, directly attack the anterior horn cells of the spinal cord. This causes a longitudinal anterior myelitis on imaging, and a poliomyelitis-like flaccid paralysis clinically.^31 32^

ZIKV-associated cases of myelitis appear to have elements of both transverse and anterior patterns, including evidence of motor, sensory and autonomic signs, 1–2 weeks after systemic symptom onset. Spinal cord lesions on MRI may be anterior and longitudinally extensive. Clinical improvement may follow standard treatment with corticosteroids or plasma exchange.^17 33 34^

### Acute disseminated encephalomyelitis

**Figure 3:**
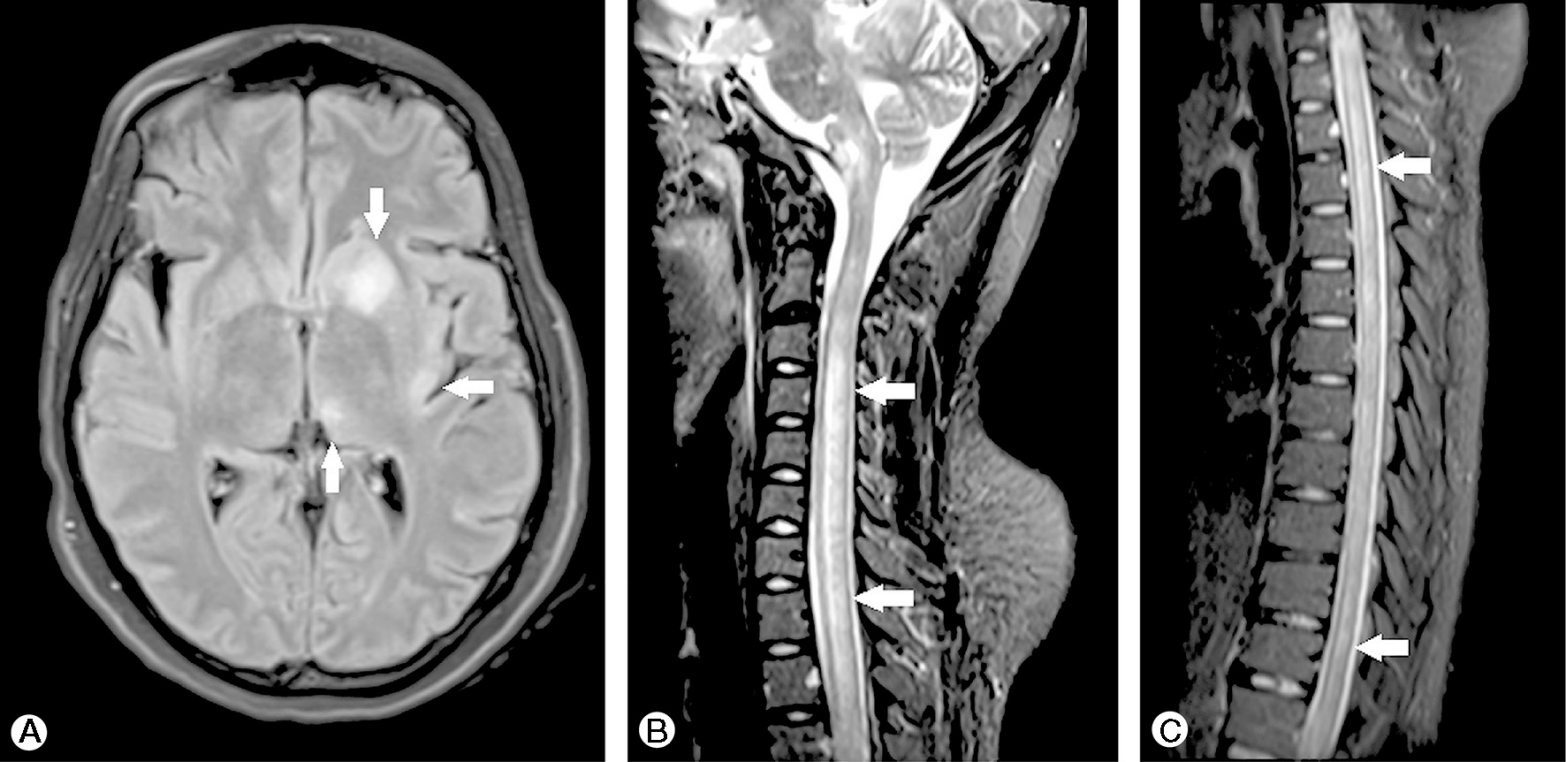
MR scan of brain fluid-attenuated inversion recovery (FLAIR) (**A**) and short tau inversion recovery (STIR) (**B**, **C**) showing asymmetrical hyperintensities affecting the left nucleocapsular region, thalamus and insula (**A**), and hyperintense longitudinally extensive lesions in the spinal cord. (Copyright © S Karger AG, Basel; Niemeyer *et al*^36^).

Acute disseminated encephalomyelitis (ADEM) is a parainfective or postinfective immunological disease associated with multifocal neurological symptoms and encephalopathy, often occurring in young adults and children as is seemingly the case with ZIKV-associated ADEM. The relationship between viral infection and disease onset is heterogeneous among reported cases and there are no known ZIKV-specific clinical or MRI features (figure 3).^35 36^

### ZIKV diagnosis in neurological disease

**Figure 4:**
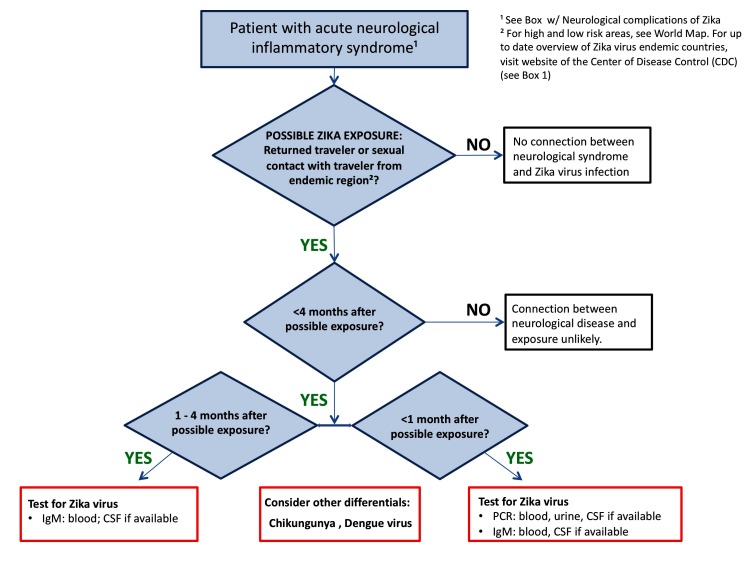
Algorithm for laboratory investigation of suspected Zika virus infection. CHIKV, chikungunya virus; CSF, cerebrospinal fluid; DENV, dengue virus.

Figure 4 gives an algorithm for laboratory investigation of suspected ZIKV infection. Recent ZIKV infection is confirmed through RT-PCR of serum, urine and CSF specimens and ZIKV IgM antibody-capture ELISA (MAC ELISA) of serum and CSF.^37^

Reliable detection of viral RNA is possible for around 14 days in serum and urine. However, as there is a variable window of time between viral infection and neurological symptoms, PCR may well be performed when viral RNA is no longer identifiable.^9^

ZIKV-specific IgM antibody of serum or CSF can be detected from around 4 days to at least 12 weeks after exposure, although tests have poor specificity due to cross-reactivity with antibodies to other structurally similar viruses such as dengue virus.^37^ More reliable testing for the presence of antibodies that neutralise the virus is possible with plaque reduction neutralisation testing but this goes beyond the capability of most hospital laboratories. Detection of virus or antibody in the CSF is more specific for CNS disease caused by ZIKV, than detection in serum, urine or semen only.

### Drugs and vaccines against ZIKV

There is no specific antiviral treatment available for ZIKV infection. Management of systemic symptoms should be supportive and standard practice should be followed for cases of ZIKV-related neurological disease.

There are several vaccines in development and two have entered phase 1 clinical trials, although their progress is complicated by multiple factors.^38^ Most importantly, vaccine safety must be guaranteed in pregnant women as they are the main population of interest, and the possibility of vaccine-induced GBS should be considered and prevented. It will likely take years before these vaccines are on the market.

## Conclusion

The rapid emergence of ZIKV as a potential cause of severe neurological disease has significant implications in endemic areas and beyond. Unsuspecting travellers and an abundance of potential vectors have facilitated its prolific spread, as illustrated by the number of imported cases to date and the extent of at-risk areas for future outbreaks. We advise neurologists working in areas without current active transmission of the virus to consider a preceding ZIKV infection in patients with acute inflammation of the central or peripheral nervous system returning from areas with ongoing active viral transmission, or with sexual partners who have returned from such areas. There are currently no antiviral drugs available for ZIKV and recommended treatment does not differ from standard practice.

**Key points**

- We advise testing for Zika virus in patients with suspected inflammatory neuropathy, unexplained myelitis or meningoencephalitis who have been in an area with local transmission or who have had sexual contact with a confirmed Zika virus case, with or without preceding viral symptoms.
- The recommended treatment in Zika virus-associated neurological disease does not differ from standard practice and there are currently no effective antiviral drugs.
- There are several vaccine candidates against Zika virus in development.
